# Shorter SPECT scans using self-supervised coordinate learning to synthesize skipped projection views

**DOI:** 10.1186/s40658-025-00762-3

**Published:** 2025-05-20

**Authors:** Zongyu Li, Yixuan Jia, Xiaojian Xu, Jason Hu, Jeffrey A. Fessler, Yuni K. Dewaraja

**Affiliations:** 1 Department of Electrical Engineering and Computer Science, University of Michigan, Ann Arbor, MI 48109-2122 USA; 2 Department of Radiology, University of Michigan, Ann Arbor, MI USA

**Keywords:** Self-supervised learning, SPECT, Sparse projection views, Lu-177 imaging

## Abstract

**Purpose:**

This study addresses the challenge of extended SPECT imaging duration under low-count conditions, as encountered in Lu-177 SPECT imaging, by developing a self-supervised learning approach to synthesize skipped SPECT projection views, thus shortening scan times in clinical settings.

**Methods:**

We developed **SpeRF**, a **SPE**CT reconstruction pipeline that integrates synthesized and measured projections, using a self-supervised coordinate-based learning framework inspired by Neural Radiance Fields (Ne**RF**). For each single scan, SpeRF independently trains a multi-layer perceptron (MLP) to estimate skipped SPECT projection views. SpeRF was tested with various down-sampling factors (DFs = 2, 4, 8) on both Lu-177 phantom SPECT/CT measurements and clinical SPECT/CT datasets, from 11 patients undergoing [177Lu]Lu-DOTATATE and 6 patients undergoing [177Lu]Lu-PSMA-617 radiopharmaceutical therapy. Performance was evaluated both in projection space and by comparing reconstructed images using (1) all measured views (“Full”), (2) down-sampled measured views only (“Partial”), and partially measured views combined with skipped views (3) generated by linear interpolation (“LinInt”) and (4) synthesized by our method (“SpeRF”).

**Results:**

SpeRF projections demonstrated lower Normalized Root Mean Squared Difference (NRMSD) compared to the measured projections, than LinInt projections. Across various DFs, the NRMSD for SpeRF projections averaged 7% vs. 10% in phantom studies, 18% vs. 26% in DOTATATE patient studies, and 20% vs. 21% in PSMA-617 patient studies, compared to LinInt projections. For SPECT reconstructions, DF = 4 is recommended as the best trade-off between acquisition time and image quality. At DF = 4, in terms of Contrast-to-Noise Ratio relative to Full, SpeRF outperformed LinInt and Partial: (1) DOTATATE: 88% vs. 69% vs. 69% for lesions and 88% vs. 73% vs. 67% for kidney, (2) PSMA-617: 78% vs. 71% vs. 69% for lesions and 78% vs. 57% vs. 67% for organs, including kidneys, lacrimal glands, parotid glands, and submandibular glands. SpeRF slightly underestimated count recovery relative to Full, compared to Partial but still outperformed LinInt: (1) DOTATATE: 98% vs. 100% vs. 88% for lesions and 98% vs. 100% vs. 94% for kidney, (2) PSMA-617: 98% vs. 101% vs. 94% for lesions and 96% vs. 101% vs. 78% for organs.

**Conclusion:**

The proposed method, SpeRF, shows potential for significant reduction in acquisition time (up to a factor of 4) while maintaining quantitative accuracy in clinical SPECT protocols by allowing for the collection of fewer projections. The self-supervised nature of SpeRF, with data processed independently on each patient’s projection data, eliminates the need for extensive training datasets. The reduction in acquisition time is particularly relevant for imaging under low-count conditions and for protocols that require multiple-bed positions such as whole-body imaging.

**Supplementary Information:**

The online version contains supplementary material available at 10.1186/s40658-025-00762-3.

## Introduction

SPECT/CT imaging has had many advances [1]; however, one continuing limitation is that SPECT acquisition is slow, especially under the low-count conditions encountered when imaging therapy radionuclides, such as Y-90, Ac-225, Ra-223, and Lu-177. These radionuclides are chosen for the therapeutic properties of their alpha and beta emissions, hence do not have ideal properties for gamma-camera imaging. For example, the photon/gamma-ray yield is relatively low, leading to low count conditions. Nevertheless, it is very desirable to perform both therapy and imaging with the same radionuclide, even in very low-count applications.

With Lu-177 where the 208 keV gamma-ray emission probability is only 10%, it can take 15–30 min per bed (~ 40 cm axial) for SPECT on standard gamma-camera systems following radiopharmaceutical therapies (RPTs) such as [177Lu]Lu-DOTATATE and [177Lu]Lu-prostate-specific membrane antigen (PSMA-617) [2]. For RPTs involving alpha-emitters, such as [225Ac]Ac-PSMA-I&T, acquisition times of up to 1 h have been proposed [3]. This is because both the administered activities and the gamma-ray yields are very low. SPECT under low-count conditions is particularly challenging when multiple beds are needed to encompass critical organs and metastases throughout the body. For example, in [177Lu]Lu-PSMA-617 therapy for metastatic castration-resistant prostate cancer (mCRPC), SPECT imaging may require up to 3 bed positions to include all critical organs such as lacrimal glands, salivary glands, bone marrow, and kidneys, as well as lesions that can be found throughout the body [4, 5]. Multi-bed position SPECT imaging demands a significant amount of camera time, which can not only lead to patient discomfort, but can also increase motion artifacts. Additionally, in many facilities, camera availability can be limited.

To overcome these challenges, a shorter acquisition time is preferable by taking either fewer projection views or shorter acquisition time per view. These strategies pose additional challenges due to either the missing (skipped) view angles or the increased image noise [6]. Numerous algorithms have been proposed with a focus on enhancing the image quality of the reconstructed images from noisy projections [7–14]. As an example, Pan et al. introduced a content-attention image restoration approach to recover high-quality images from low-dose planar bone scans obtained during fast acquisitions [15]. In contrast, the approach of synthesizing the missing projections [2, 16] has been relatively unexplored. Most prior studies have employed deep learning techniques to learn the relationship between one projection and its neighboring views, often relying on ground truth data for training purposes. For instance, Rydén et al. trained a deep convolutional U-Net [17] to generate synthetic intermediate projections [2, 18]. Meanwhile, Li et al. introduced a network architecture called LU-Net that integrates Long Short-Term Memory network [19] and U-Net to understand the transformation from sparse-view projection data to full-view data [20]. Chen et al. presented a cross-domain method using SPECT images predicted in the image domain as reference for synthesizing full-view projections in the sinogram domain [21]. These approaches are reported to be effective, but they are all based on supervised learning methods that require hundreds of paired data for training. However, in many cases, obtaining enough paired ground truth data for training is challenging. This difficulty is especially true in the case of post-therapy imaging for verifying uptake or dosimetry following RPT because such imaging is typically not part of routine clinical practice in some countries. On the other hand, self-supervised learning, which does not require separate training labels and instead learns from each scan itself, has the potential to overcome the limitations of supervised learning in such scenarios.

The aim of this research was to reduce SPECT acquisition time by reducing the required number of measured projection views while maintaining image quality by incorporating synthetic projections generated by deep neural networks. We implemented a multi-layer perceptron (MLP) and trained it to generate skipped SPECT projection views through self-supervised coordinate learning [22]. We evaluated the performance of the proposed method both qualitatively and quantitatively in phantom studies and in patients imaged after [177Lu]Lu-DOTATATE therapy and [177Lu]Lu-PSMA-617 therapy.

## Materials and methods

### Phantom study

We used an elliptical phantom with six hot sphere inserts of volumes 2,4,8,16,30,114mL. These ‘hot’ spheres (having the same Lu-177 activity concentration of 0.22 MBq/mL) were placed in a ‘warm’ background (0.035 MBq/mL) to achieve a sphere-to-background ratio of 6.3:1, which is representative of tumor-to-background ratios encountered in patient imaging [23, 24]. The total activity in the phantom at scan time was 356 MBq. The sphere volumes of interest (VOIs), corresponding to the physical filling volume, were defined on the CT images.

### Patient studies

We used retrospective SPECT/CT data from patients who had volunteered for imaging under a University of Michigan Institutional Review Board (IRB) approved protocol. This included 11 patients imaged after [177Lu]Lu-DOTATATE therapy for neuroendocrine tumor (NET) and 6 patients imaged after [177Lu]Lu-PSMA-617 therapy for mCRPC. We defined organs of interest (kidneys for DOTATATE therapy, and kidneys, lacrimal glands, parotid glands, and submandibular glands for [177Lu]Lu-PSMA-617 therapy) using deep learning-based segmentation methods available within MIM Software^®^. A radiologist manually defined the lesions (78 in total, volume ranging from 2 to 250 mL) as described previously [24].

### SPECT/CT acquisition

All scans were acquired on a Siemens Intevo Bold SPECT/CT with a 5/8’’ crystal equipped with medium-energy low penetration (MELP) collimators. Acquisition parameters included 120 views, with 60 views per head, a 20% photopeak window centered at 208 keV, and two adjacent scatter windows of 10% width each. The phantom study used a prolonged acquisition of 196 s/view to achieve a count level which is representative of patient imaging after the administration of [177Lu]Lu-DOTATATE. The patient images were acquired under the standard protocols used in our clinic. [177Lu]Lu-DOTATATE SPECT images were acquired for a single bed position at day 2 or day 4 after the cycle 1 administration of 7.4 GBq using an acquisition time of 25 s per view (total scan time of 25 min). The [177Lu]Lu-PSMA-617 SPECT images were acquired with two bed positions at day 2 or day 3 after the cycle 1 administration of 7.4 GBq with an acquisition time of 17 s per view per bed (total scan time of 34 min). The projection view matrix size was 128 × 128, with a pixel size of 4.8 × 4.8 mm. The CT images, with a matrix size of 512 × 512 and a pixel size of 0.98 × 0.98 mm, were acquired in low-dose mode (120 kVp; 15–80 mAs) under free-breathing conditions. The slice thickness was 3 mm for [177Lu]Lu-DOTATATE patients and 1.5 mm for [177Lu]Lu-PSMA-617 patients.

### Self-supervised coordinate learning

Given the limited amount of data, we focused on a self-supervised learning approach, namely SpeRF, rather than supervised methods for this study. Our method is inspired by the neural radiance field (NeRF) approach that maps 3D spatial coordinates to radiance values using a neural network [25]. Similarly, we developed a coordinate-based MLP with 12 hidden layers and 256 neurons per layer to synthesize skipped projection views in SPECT imaging.

#### Network framework and workflow

**Fig. 1:**
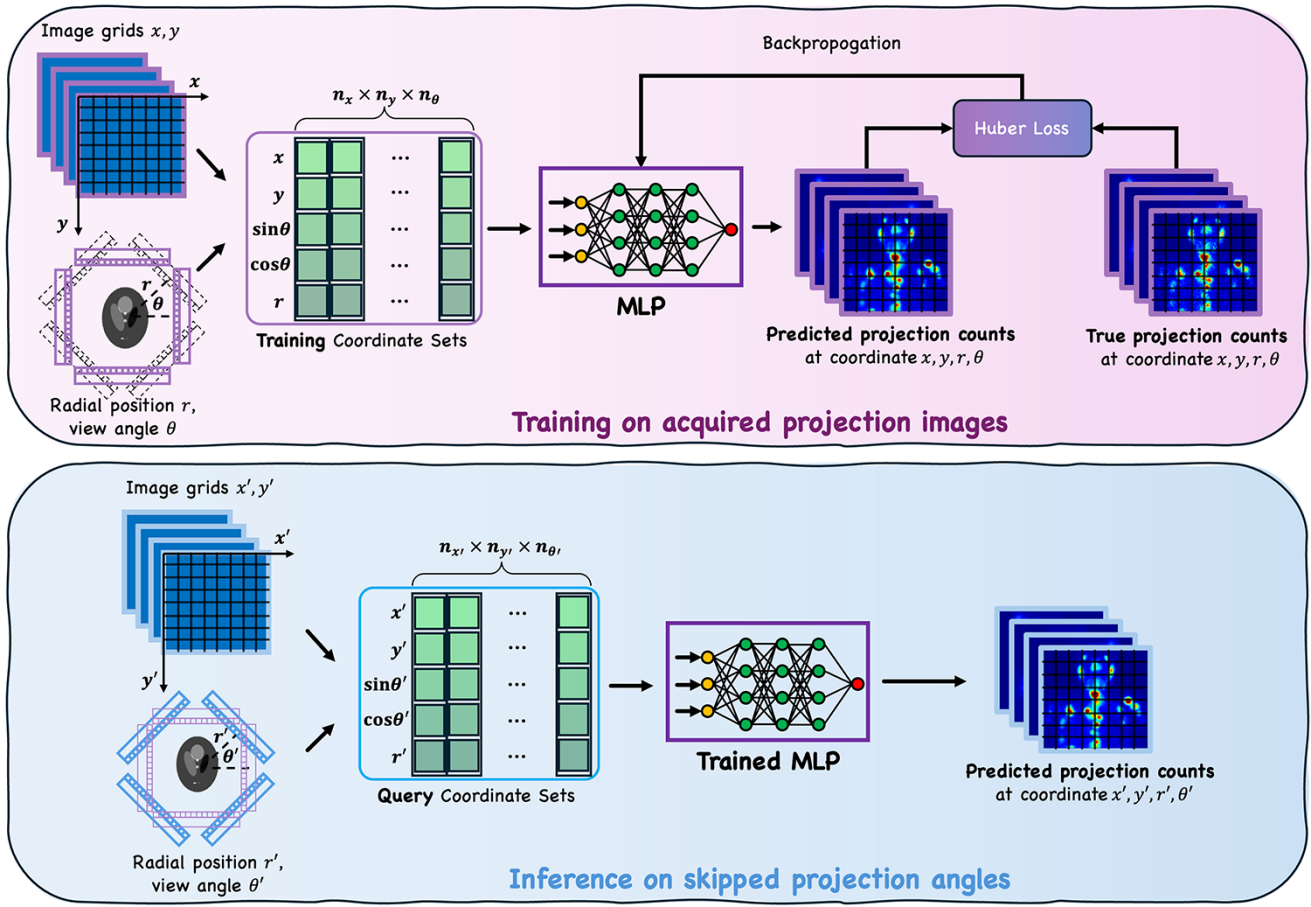
Overview of SpeRF pipeline. The top panel (light purple) illustrates the training phase, where a coordinate-based MLP processes training coordinate sets consisting of $\:x,\:y$ coordinates, radial position $\:r$, and trigonometric features $\:sin\theta\:\:$ and $\:cos\theta\:$ derived from the view angle $\:\theta\:$. The MLP predicts projection counts at the corresponding coordinates, which are compared to true projection counts using the Huber Loss function for backpropagation. The bottom panel (blue) shows the inference phase, where the trained MLP processes query coordinate sets corresponding to skipped projection angles. This allows the model to interpolate or predict projection counts at unseen angles that were not part of the training data. SpeRF is a patient-specific method, where a separate MLP is trained and used for each patient’s dataset to account for individual imaging characteristics and variations. The same MLP architecture is used in both phases, but training is performed on known projections, while inference generates skipped projections

The core of our method is a coordinate-based MLP that operates on 5-dimensional input coordinates for each pixel in the SPECT projection views (Fig. 1). These coordinates include: (1) pixel positions $\:(x,\:\:y)$, (2) the sine and cosine of the view angle $\:(\text{s}\text{i}\text{n}\theta\:,\:\:\text{c}\text{o}\text{s}\theta\:)$, and (3) the radial position $\:r$, which accounts for noncircular orbits. The MLP processes each 5D coordinate independently, predicting a single scalar projection count for that pixel. While the input coordinate set for all projection views are conceptually of size $\:{n}_{x}$×$\:{n}_{y}$×$\:{n}_{\theta\:}$×5, the network operates efficiently by iterating over individual 5D inputs, producing outputs of size $\:{n}_{x}$×$\:{n}_{y}$×$\:{n}_{\theta\:}$×1. Here, $\:{n}_{x}$ and$\:\:{n}_{y}$ represent the projection matrix dimensions, and $\:{n}_{\theta\:}$ denotes number of projection view angles, which correspond to the measured angles during training and the skipped angles during inference.

During training, the MLP learns to map the input training coordinate sets to the measured projection counts. To ensure patient-specific learning, separate MLPs are trained for each patient using their available measured projections. Additionally, for each patient, we train two independent MLPs: one for the main acquisition window and the other for the sum of the two scatter windows.

In the inference phase, the trained MLP is provided with the query coordinate sets corresponding to the skipped projection angles. The network predicts the projection counts for each pixel at these missing angles, enabling the synthesis of skipped projections. This coordinate-based design provides the flexibility to accommodate various down-sampling factors (DFs). For example, with 30 measured views and 90 synthesized views, SpeRF achieves a 75% reduction in scan time (DF = 4) while maintaining the same network architecture and hyperparameters across different DFs.

#### Rescale trick

To enhance the representation of the continuous measurement field, we applied the ‘rescale trick’, i.e., upscaling the original 128 × 128 projection images to 256 × 256 using nearest neighbor interpolation during the input stage. During training, the measured projection counts at 256 × 256 resolution serve as the target. After inference, the synthesized 256 × 256 projections are downscaled back to 128 × 128 using the same nearest neighbor interpolation method to align with the original resolution for reconstruction. Nearest neighbor interpolation duplicates the closest pixel value into the upscaled grid, preserving the original pixel intensities. After inference, the results are downscaled back to 128 × 128 by selecting (adhered to a pre-defined rule) one pixel per 2 × 2 block. This strategy maintains the pixel intensity distribution while allowing the network to operate on a finer spatial grid. Although no new information is introduced, the higher resolution helps the network approximate a smoother and more continuous measurement field, capturing essential sharp edges and variations of projection images.

### Training and optimization

For each scan, we optimized the MLP weights by minimizing the Huber loss function, defined as

$$\:{L}_{\delta\:}\left(a\right)=\left\{\begin{array}{c}\frac{1}{2}{a}^{2}\\\:\delta\:\bullet\:(\left|a\right|-\frac{1}{2}\delta\:)\end{array}\:\right.\begin{array}{c}\:\text{\:for\:}\left|a\right|<\delta\:\\\:\text{\:otherwise}\end{array}$$

Here, $\:a$ represents the difference between the MLP-predicted projection count and the measured projection count at a corresponding coordinate, and $\:\delta\:$ is a hyperparameter that controls the transition between the squared loss and the absolute loss. The Huber loss is particularly suited for this task because it is robust to outliers, which are common in noisy measurements. It combines aspects of two common loss functions: for smaller errors ($\:\left|a\right|<\delta\:$), it behaves like Mean Squared Error (MSE) providing strong learning signals due to its differentiability; for larger discrepancies ($\:\left|a\right|\ge\:\delta\:$), it behaves like Mean Absolute Error (MAE), making it less sensitive to outliers, as demonstrated in [26]. We empirically set $\:\delta\:=1$ in our implementation.

To optimize the MLP, we minimized the Huber loss function using the Adam optimizer [27] with an initial learning rate of 0.001. A reduce-on-plateau scheduler was applied to dynamically lower the learning rate when the validation loss plateaued. For each scan, we randomly selected 20% of the pixel coordinates and their corresponding projection counts from the available measured projection views as validation data. The final patient-specific model was selected based on the lowest validation loss over 200 training epochs. We used a batch size of 10,000 coordinates sampled from the $\:256\times\:256\times\:{n}_{\text{bed}}\times\:{n}_{\theta\:}$ input space. During inference, skipped projections were synthesized in approximately 40 s per view on a system equipped with a single RTX 4090 GPU, 64 GB of DDR5 memory, and a 24-core Intel i9-13900KF CPU. The implementation of our method, including training and testing virtual patient phantom images, is publicly available in PyTorch at: https://github.com/ZongyuLi-umich/.

### SPECT reconstruction

The ordered-subset expectation-maximization (OS-EM) algorithm [28] is commonly used to reconstruct 3D SPECT images. In this study we performed OS-EM SPECT reconstructions ([177Lu]Lu-DOTATATE matrix size: 128 × 128 × 81 and 2-bed [177Lu]Lu-PSMA-617 matrix size: 128 × 128 × 158, both with voxel size in mm: 4.8 × 4.8 × 4.8) with 6 subsets and 16 iterations using an in-house open-sourced toolbox, benchmarked on CPU with multi-threading and verified by Monte Carlo simulation [29]. No post-processing filter was applied. Scatter correction was applied using a triple energy window method, and attenuation correction was based on the standard CT-to-density calibration curve. The point spread function for depth-dependent collimator-detector response modeling was simulated with Monte Carlo [30] using a point source in air and fitted with Gaussian curves.

### Evaluation

SPECT image quality was evaluated for four distinct data processing pipelines: (1) “Full”: OS-EM reconstruction using all 120 measured projections. (2) “Partial”: OS-EM reconstruction using a certain DF of the measured projections. (3) “LinInt”: a certain DF of projections were measured, and the remaining projections were generated through linear interpolation, followed by OS-EM. (4) “SpeRF”: a certain DF of projections were measured, and the remaining were MLP-predicted synthetic projections, followed by OS-EM.

Our evaluation was structured into three aspects: Synthesized Projections, Phantom Reconstructions, and Patient Reconstructions, each with different metrics. When comparing synthesized projections (from SpeRF and LinInt) against measured projections for both phantom and patient studies, we used the Normalized Root Mean Squared Difference (NRMSD) defined as:

$$\:\text{N}\text{R}\text{M}\text{S}\text{D}=\:\frac{\sqrt{\frac{1}{{n}_{p}}\sum\:_{j=1}^{{n}_{p}}{({\widehat{x}}_{j}-{x}_{j})}^{2}}}{\sqrt{\frac{1}{{n}_{p}}\sum\:_{j=1}^{{n}_{p}}{x}_{j}^{2}}},$$

where $\:{n}_{p}$ is the total number of voxels within the VOI, including lesions and relevant organs. Subscript $\:j$, i.e., $\:{x}_{j}$, denotes the $\:j$th voxel in the image. The reference image and the reconstructed image are denoted by $\:x$ and $\:\widehat{x}$, respectively.

When evaluating reconstructions in phantom studies, we calculated the noise level and the Activity Recovery (AR) to assess how well the reconstruction matched the true activity map. The background (BKG) was defined as the union of six uniform “warm” regions, ensuring no overlap with the hot sphere VOIs. The noise level was computed as the standard deviation of voxel activity within this BKG, denoted as $\:{\text{S}\text{T}\text{D}}_{\text{B}\text{K}\text{G}}$. The AR is defined as:

$$\:\text{A}\text{R}=\:\frac{{\text{m}\text{e}\text{a}\text{n}(\text{r}\text{e}\text{c}\text{o}\text{n}\_\text{a}\text{c}\text{t}\text{i}\text{v}\text{i}\text{t}\text{y}}_{\text{V}\text{O}\text{I}})}{{\text{m}\text{e}\text{a}\text{n}(\text{t}\text{r}\text{u}\text{e}\_\text{a}\text{c}\text{t}\text{i}\text{v}\text{i}\text{t}\text{y}}_{\text{V}\text{O}\text{I}})}.$$

When evaluating reconstructions in patient studies, we defined relative measures, including Relative Count Recovery (RCR) and Relative Contrast-to-Noise Ratio (RCNR), in comparison to the Full recon. Here, the BKG was chosen as a homogeneous region within the lung. The RCNR and RCR are defined as:

$$\:\text{C}\text{N}\text{R}=\frac{{\text{m}\text{e}\text{a}\text{n}(\text{r}\text{e}\text{c}\text{o}\text{n}\_\text{c}\text{o}\text{u}\text{n}\text{t}}_{\text{V}\text{O}\text{I}})-{\text{m}\text{e}\text{a}\text{n}(\text{r}\text{e}\text{c}\text{o}\text{n}\_\text{c}\text{o}\text{u}\text{n}\text{t}}_{\text{B}\text{K}\text{G}})}{{\text{S}\text{T}\text{D}}_{\text{B}\text{K}\text{G}}}$$

$$\:\text{R}\text{C}\text{N}\text{R}=\frac{{\text{C}\text{N}\text{R}}_{\text{s}\text{p}\text{a}\text{r}\text{s}\text{e}\_\text{v}\text{i}\text{e}\text{w}\:\text{r}\text{e}\text{c}\text{o}\text{n}}}{{\text{C}\text{N}\text{R}}_{\text{F}\text{u}\text{l}\text{l}\_\text{r}\text{e}\text{c}\text{o}\text{n}}\:}\times\:100\%$$

$$\:\:\:\text{R}\text{C}\text{R}=\frac{{\text{m}\text{e}\text{a}\text{n}(\text{s}\text{p}\text{a}\text{r}\text{s}\text{e}\_\text{v}\text{i}\text{e}\text{w}\_\text{r}\text{e}\text{c}\text{o}\text{n}\_\text{c}\text{o}\text{u}\text{n}\text{t}}_{\text{V}\text{O}\text{I}})}{{\text{m}\text{e}\text{a}\text{n}(\text{F}\text{u}\text{l}\text{l}\_\text{r}\text{e}\text{c}\text{o}\text{n}\_\text{c}\text{o}\text{u}\text{n}\text{t}}_{\text{V}\text{O}\text{I}})}\times\:100\%,\:\:$$

## Results

### Synthesized projections

Table 1 compares the performance of linearly interpolated projections against SpeRF projections, summarizing the NRMSD values across various DFs for phantom studies and patient studies. The results consistently demonstrate that the SpeRF projections outperform LinInt projections, exhibiting lower NRMSD values in both phantom and patient studies.

**Fig. 2:**
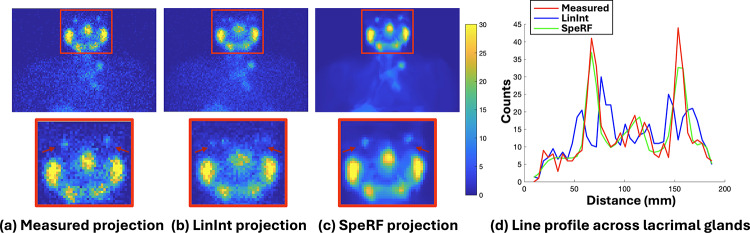
Comparison of measured and synthesized projections for a patient after [177Lu]Lu-PSMA-617 therapy. Fig. (**a**), (**b**), and (**c**) show measured projection, LinInt projection (generated through linear interpolation), and SpeRF projection, respectively. The images and profile comparison across lacrimal glands show two hot spots/peaks in the SpeRF projection (green line) corresponding to left and right lacrimals, closely resembling the profile of the measured projection (red line), whereas the corresponding LinInt projection profile shows 4 peaks due to distortions caused by angular interpolation

Visually, SpeRF projections appear smoother than their measured counterparts. Figure 2 displays the measured (Fig. 2a) and synthesized projections (Fig. 2b and c) for a representative [177Lu]Lu-PSMA-617 patient. Close examination of the intensity profiles across the lacrimals reveals notable differences: the SpeRF projection exhibits two peaks (corresponding to high uptake in left and right lacrimals as expected with [177Lu]Lu-PSMA-617), more closely aligning with the pattern observed in the measured projection, while the LinInt projection presents four peaks due to angular interpolation.

**Table 1:** NRMSD comparisons between sperf projections and LinInt projections, relative to measured projections, across different down-sampling factors (DFs) for Phantom study and patient studies (values are average across 11 [177Lu]Lu-DOTATATE studies and 6 [177Lu]Lu-PSMA-617 studies)

	Phantom Study		Patient Study			
			DOTATATE		PSMA-617	
	SpeRF	LinInt	SpeRF	LinInt	SpeRF	LinInt
**DF = 2**	**5.9%**	9.0%	**16.9%**	23.4%	**17.5%**	24.6%
**DF = 4**	**6.2%**	9.5%	**17.5%**	25.5%	**18.4%**	27.4%
**DF = 8**	**7.5%**	11.1%	**18.8%**	30.4%	**23.7%**	34.1%

### Phantom reconstruction results

**Fig. 3:**
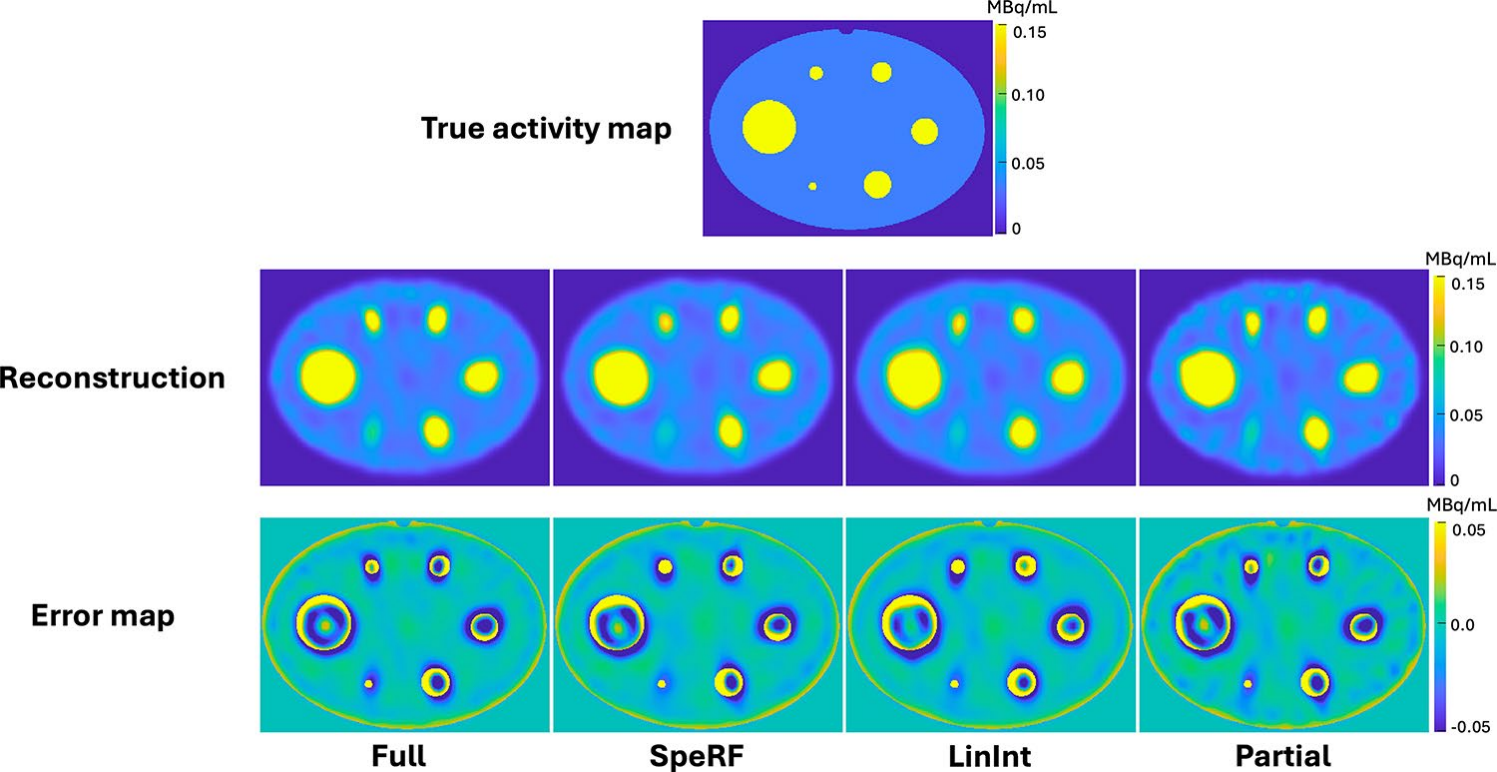
Visual comparison of Full recon, SpeRF recon, LinInt recon and Partial recon, against phantom true activity, for DF = 4. All reconstructed images and true activity maps are in the same color scale. Error maps present pixel value differences between reconstructed images and true activity

**Fig. 4:**
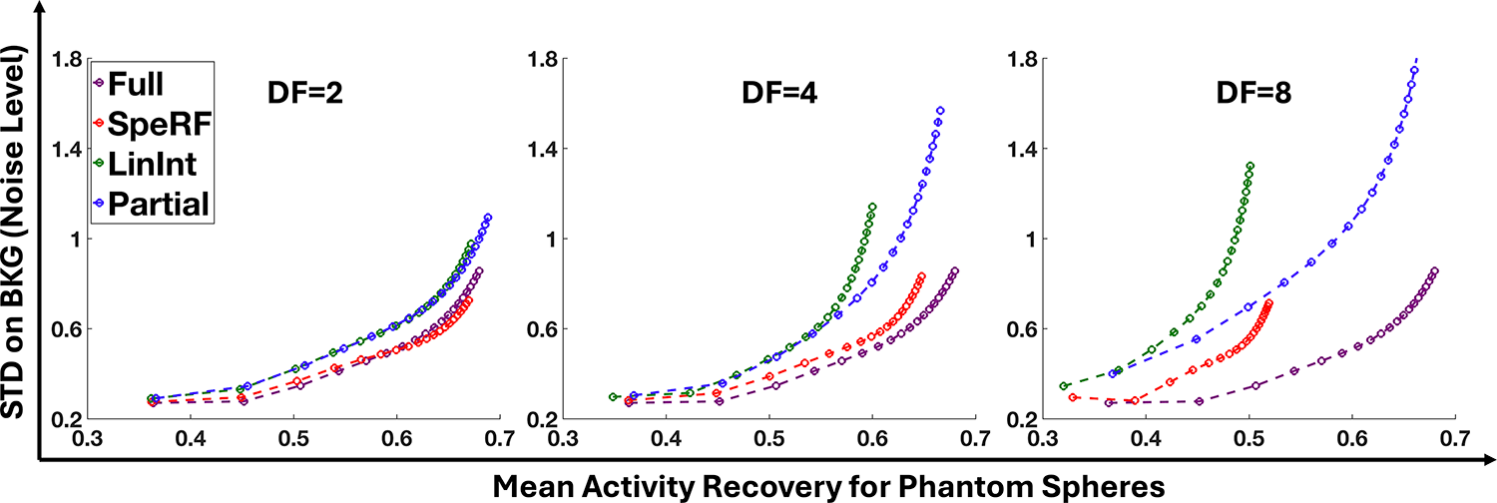
Noise to mean activity recovery (AR) curves averaged across six sphere volumes for DFs = 2, 4, and 8 are shown in subplots (**a**), (**b**), and (**c**), respectively. When DF = 2 and 4, SpeRF recon consistently outperforms both LinInt and Partial recon, achieving noise-to-AR performance that most closely aligns with Full recon across OS-EM iterations. When DF = 8, Partial recon exhibits significantly increased noise level, while both SpeRF and LinInt suffer from reduced sphere AR

Consider the DF = 4 scenario as an illustrative case. Figure 3 compares four data processing pipelines (Full, SpeRF, LinInt, Partial) with the true activity map. Although each pipeline exhibits structural similarities with the true activity, the Partial recon is noticeably noisier than its counterparts. Quantitative comparisons, presented in Fig. 4, plot noise to mean activity recovery (average across all six hot spheres) curves at DF = 2, 4 and 8, where the SpeRF recon outperforms both the Partial recon and LinInt recon by most closely paralleling the Full recon through OS-EM iterations. Note that even for the Full recon, AR is degraded (AR < 1) due to the partial volume effects [31]. Supplementary Fig. 1 provides individual noise to activity recovery curves for each hot sphere.

Moreover, the noise level in all sparse-view reconstructions increases as the DF becomes larger. But the SpeRF recon consistently achieves highest activity recoveries for all six lesions at the same noise level. When DF = 8, as evident in Fig. 4(c), the Partial recon attained higher activity recovery for small lesions, at the expense of substantially increased noise level, while the SpeRF recon remains superior for larger lesions. For all sizes of lesions and DFs, the SpeRF recon matched the activity recovery of the LinInt recon while maintaining a significantly lower noise level.

### Patient reconstruction results

**Fig. 5:**
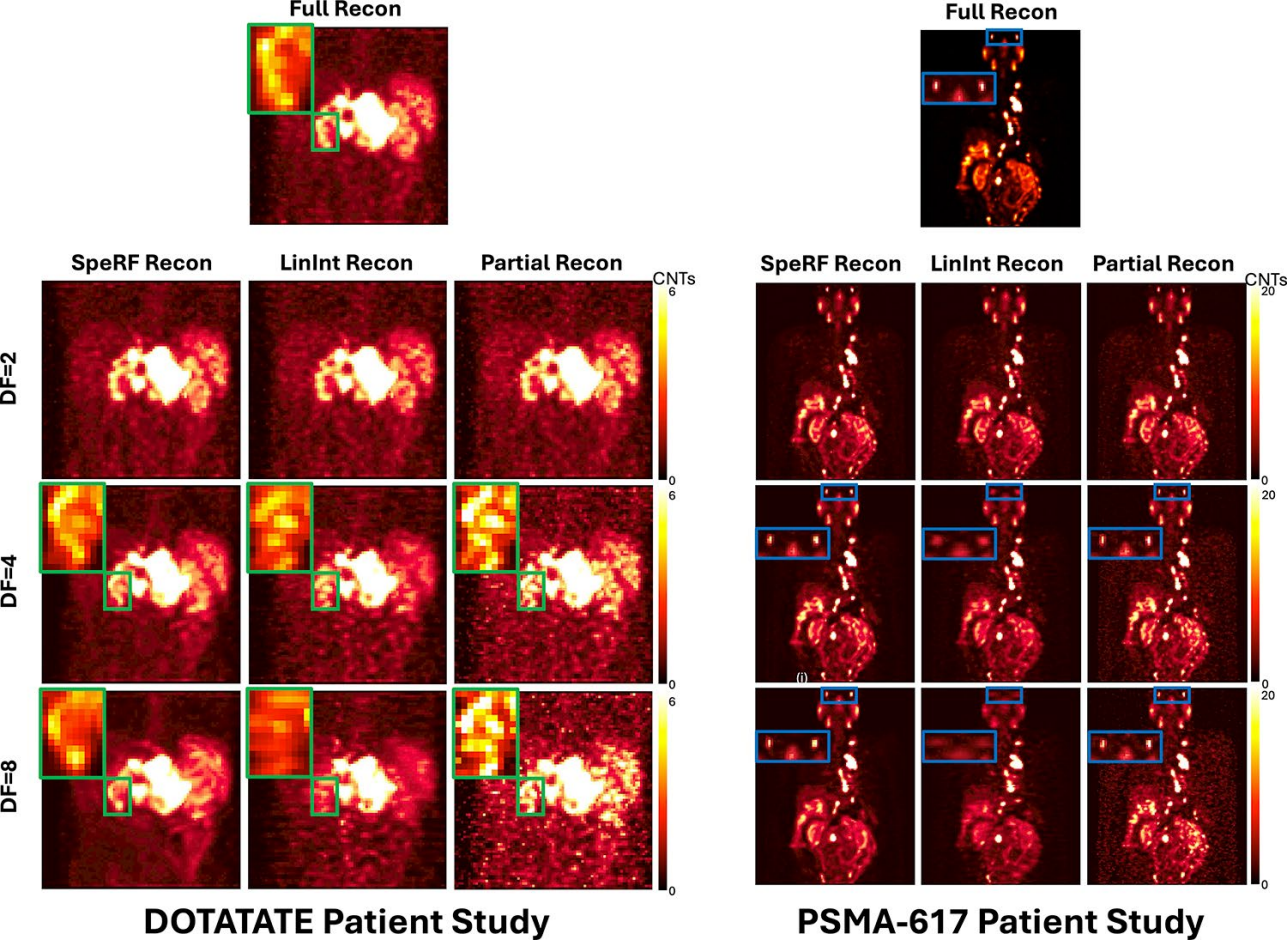
Coronal Maximum Intensity Projections of SPECT reconstructions for [177Lu]Lu-DOTATATE (left panel) and [177Lu]Lu-PSMA-617 (right panel) patient studies are presented side by side, using four data processing pipelines (columns) and three DFs (rows). Colored boxes highlighted regions with apparent distinctions, which are zoomed in for closer inspection. Gamma correction, a non-linear adjustment of displayed pixel intensities, is applied to all images to enhance contrast and emphasize reconstruction artifacts. In both panels, blurring artifacts in the LinInt recon and noise in the Partial recon become increasingly prominent, especially obvious at higher DFs. SpeRF recon, however, maintains image quality closer to Full recon

Figure 5 shows the coronal Maximum Intensity Projections (MIPs) of an example patient image after [177Lu]Lu-DOTATATE (Fig. 5 left) and [177Lu]Lu-PSMA-617 (Fig. 5 right) therapy, respectively, derived from four different data processing pipelines at various DFs. In both the [177Lu]Lu-DOTATATE and [177Lu]Lu-PSMA-617 studies, the LinInt recons exhibit noticeable artifacts due to distortions caused by angular interpolation, more pronounced at higher DFs. This effect is particularly evident in the [177Lu]Lu-PSMA-617 study for organs like the lacrimal, parotid, and submandibular glands at DF = 4 and 8, substantially affecting the structural clarity of the SPECT images. Partial recons became noisier with increasing DFs, making it challenging to distinguish small hot spots from the background. However, SpeRF recon maintains an image quality closer to Full recon.

**Fig. 6:**
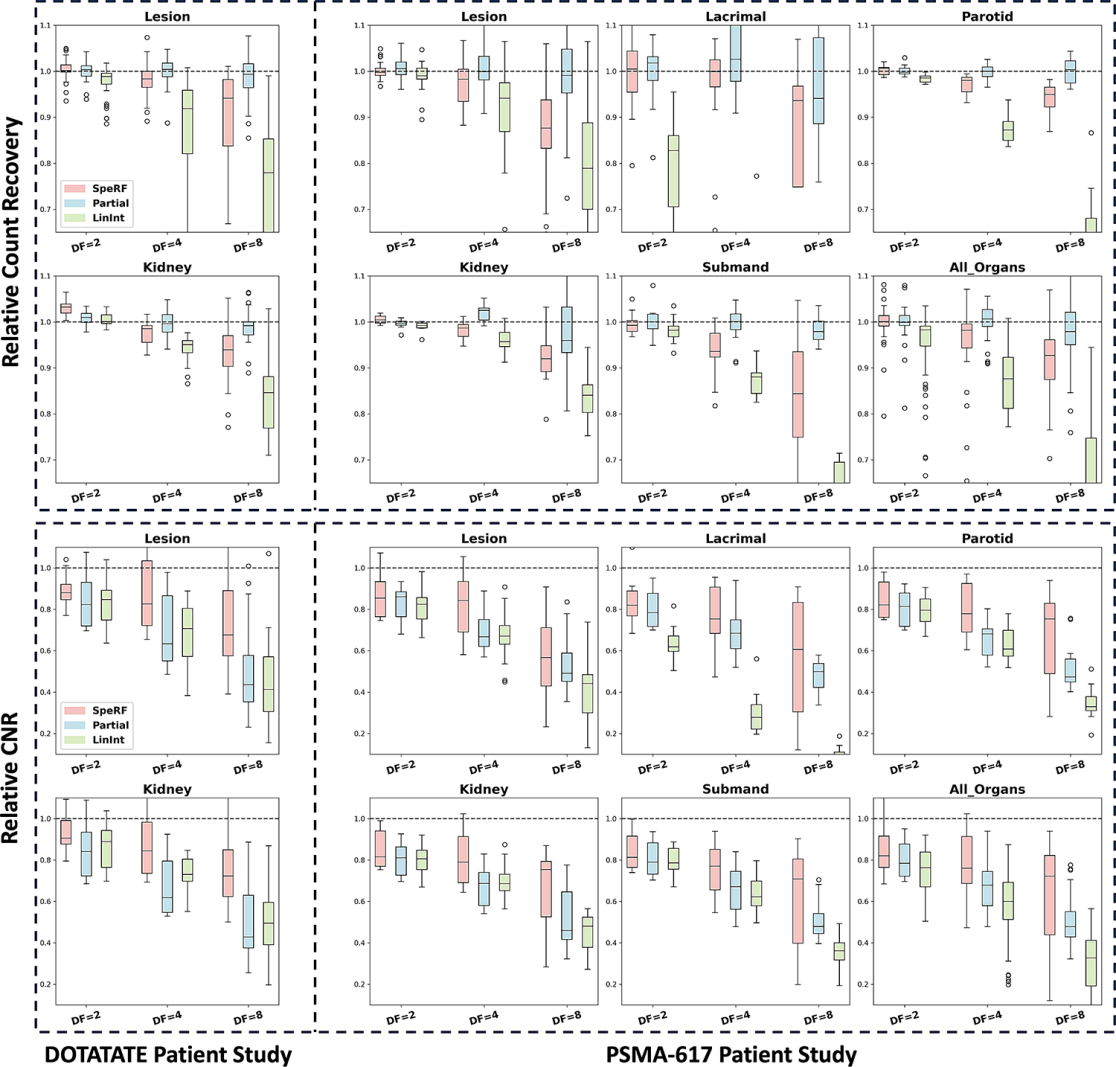
Box plot visualization of results presented in Tables 2, 3, 4 and 5, showing RCR, i.e., relative count recovery (upper panel) and relative CNR (lower panel) for three sparse-view reconstructions: SpeRF recon, Partial recon, and LinInt recon. Results are reported for [177Lu]Lu-DOTATATE (left panel) and [177Lu]Lu-PSMA-617 (right panel) patient studies across different down-sampling factors (DF = 2, 4, 8). SpeRF recon maintains a balance between RCR and RCNR (especially at DF = 4), outperforming LinInt in both metrics and Partial in RCNR. Note that for the RCR of lacrimal glands in LinInt recon, a few data points are not visible because they fall below the plotted range

The RCR and RCNR results are presented in Tables 2, 3, 4 and 5 and visualized in Fig. 6. For RCR, Partial recon consistently achieves the highest values, closely matching the Full recon (100%). SpeRF recon slightly underestimates RCR compared to Partial but remains close to Full recon and consistently outperforms LinInt recon. For example, in the [177Lu]Lu-DOTATATE study at DF = 4 (Table 2), SpeRF recon achieves RCR values of ~ 98.3% for lesions, compared to ~ 100.3% for Partial recon and ~ 87.5% for LinInt recon. Similarly, in the [177Lu]Lu-PSMA-617 study at DF = 4 (Table 3), SpeRF recon achieves ~ 98.4% RCR for lesions, slightly lower than Partial recon (~ 100.5%) but significantly better than LinInt recon (~ 93.7%). For RCNR, SpeRF recon demonstrates a consistent advantage across all DFs, particularly at DF = 4, where it achieves the best balance of RCR and RCNR. In the [177Lu]Lu-DOTATATE study at DF = 4 (Table 4), SpeRF recon achieves ~ 87.9% RCNR for lesions, outperforming both LinInt recon (~ 68.7%) and Partial recon (~ 68.7%). A similar trend is observed in the [177Lu]Lu-PSMA-617 study (Table 5), where SpeRF recon achieves ~ 78.4% RCNR for lesions at DF = 4, compared to ~ 70.7% for LinInt recon and ~ 68.5% for Partial recon. At higher DFs, such as DF = 8, SpeRF recon continues to outperform the other methods in RCNR, although the RCR decreases more significantly. These results indicate that SpeRF recon maintains high activity recovery while providing additional advantages in RCNR.

Overall, DF = 4 provides the best trade-off for SpeRF recon, offering high RCR (within ~ 2-3% of Full recon) and the highest RCNR across all VOIs. While Partial recon achieves slightly higher RCR values, SpeRF recon delivers superior RCNR, particularly in small or challenging regions such as the lacrimal glands (~ 0.4 mL), where LinInt recon often fails. These results highlight the potential of SpeRF recon as a robust method for reducing scan time while preserving image quality and clinical usability.

**Table 2:** Average relative count recovery (RCR) values of the SpeRF recon, the LinInt recon, and the Partial recon across all eleven [177Lu]Lu-DOTATATE patient studies, benchmarked against the Full recon, whose RCR is standardized at 100%

	DF = 2			DF = 4			DF = 8		
	SpeRF Recon	LinInt Recon	Partial Recon	SpeRF Recon	LinInt Recon	Partial Recon	SpeRF Recon	LinInt Recon	Partial Recon
**Lesion**	100.6%	97.7%	**100.1%**	98.3%	87.5%	**100.3%**	90.3%	71.6%	**98.8%**
**Kidney**	103.9%	100.5%	**100.8%**	97.7%	94.2%	**99.5%**	93.0%	83.7%	**99.0%**

**Table 3:** Average RCR values of the SpeRF recon, the LinInt recon, and the Partial recon across all six [177Lu]Lu-PSMA-617 patient studies, benchmarked against the Full recon, whose RCR is standardized at 100%

	DF = 2			DF = 4			DF = 8		
	SpeRF Recon	LinInt Recon	Partial Recon	SpeRF Recon	LinInt Recon	Partial Recon	SpeRF Recon	LinInt Recon	Partial Recon
Lesion	101.1%	**99.9%**	100.9%	98.4%	93.7%	**100.5%**	95.6%	92.2%	**104.5%**
All Organ ROIs	**99.9%**	93.8%	**99.9%**	96.1%	78.1%	**101.1%**	87.3%	58.8%	**97.8%**
Kidney	100.5%	99.1%	**99.6%**	**98.3%**	95.9%	102.1%	92.4%	83.0%	**98.1%**
Lacrimal	99.4%	79.6%	**99.8%**	95.5%	41.8%	**103.5%**	79.6%	21.4%	**94.9%**
Parotid	**100.2%**	98.4%	**100.2%**	97.1%	87.5%	**99.7%**	93.9%	65.6%	**100.0%**
Submandibular	99.6%	98.1%	**100.2%**	93.6%	87.3%	**99.1%**	83.3%	65.4%	**98.2%**

**Table 4:** Average relative contrast-to-noise ratio (RCNR) values of the SpeRF recon, the LinInt recon, and the Partial recon across all eleven [177Lu]Lu-DOTATATE patient studies, benchmarked against the Full recon, whose RCNR is standardized at 100%

	DF = 2			DF = 4			DF = 8		
	SpeRF Recon	LinInt Recon	Partial Recon	SpeRF Recon	LinInt Recon	Partial Recon	SpeRF Recon	LinInt Recon	Partial Recon
**Lesion**	**88.6%**	82.5%	82.7%	**87.9%**	68.7%	68.7%	**73.5%**	43.9%	48.2%
**Kidney**	**92.6%**	85.8%	84.5%	**88.0%**	73.1%	67.0%	**76.5%**	51.3%	48.8%

**Table 5:** Average RCNR values of the SpeRF recon, the LinInt recon, and the Partial recon across all six [177Lu]Lu-PSMA-617 patient studies, benchmarked against the Full recon, whose RCNR is standardized at 100%

	DF = 2			DF = 4			DF = 8		
	SpeRF Recon	LinInt Recon	Partial Recon	SpeRF Recon	LinInt Recon	Partial Recon	SpeRF Recon	LinInt Recon	Partial Recon
**Lesion**	**83.8%**	79.8%	80.7%	**78.4%**	70.7%	68.5%	**65.7%**	55.7%	54.9%
**All Organ ROIs**	**84.7%**	75.7%	80.9%	**78.4%**	56.9%	67.3%	**63.2%**	31.0%	50.8%
**Kidney**	**84.8%**	79.9%	80.3%	**80.1%**	69.6%	67.6%	**65.8%**	44.2%	51.3%
**Lacrimal**	**83.6%**	63.6%	80.4%	**77.5%**	29.9%	68.6%	**57.2%**	10.2%	47.9%
**Parotid**	**84.5%**	79.3%	80.9%	**79.1%**	63.4%	66.0%	**67.6%**	34.7%	52.0%
**Submandibular**	**85.6%**	79.7%	81.8%	**77.1%**	64.0%	66.9%	**62.2%**	34.6%	51.7%

## Discussion

The field of machine learning, particularly deep learning (DL), is rapidly advancing. However, DL applications in SPECT imaging remain limited, partly due to challenges such as limited availability of training data. Supervised DL methods, like 3D U-Net, have shown promise in predicting missing SPECT projection views, but their reliance on large datasets poses a barrier. Although simulated SPECT projection data can be useful for training, there is a domain gap between simulated data and real patient data, with differences in activity distributions and noise characteristics. This gap can limit the applicability of supervised models trained solely on simulations. In our RPT application, with only tens of patient datasets available, obtaining the hundreds or thousands of datasets required for supervised methods was impractical. Additionally, variations in camera-specific parameters, such as gamma-camera crystal thickness and body contour orbits, can hinder generalizability. In contrast, self-supervised learning methods derive insights directly from the available data, without the need for extensive labeled datasets, making them inherently adaptable. This motivated our focus on a self-supervised approach in this study.

To evaluate the performance of supervised methods with our limited dataset, we implemented a supervised learning approach similar to that introduced in [2]. Specifically, we used three separate 3D U-Nets to predict the skipped 3/4 of total projections given the 1/4 measured projections. Each U-Net was trained to predict a subset of skipped projections: the first U-Net targeted projections 2, 6, 10,…, 118; the second targeted projections 3, 7, 11,…, 119; and the third targeted projections 4, 8, 12,…, 120. We split our dataset of 11 [177Lu]Lu-DOTATATE patients into 4 for training, 2 for validation, and 5 for testing. The results, shown in Table 6, indicate that this supervised approach achieved a substantially lower RCR and RCNR compared to SpeRF (Tables 2, 3, 4 and 5), emphasizing the challenges of applying supervised methods to scenarios with restricted data availability.

**Table 6:** Average RCR and RCNR values for lesions and kidneys achieved by a supervised learning method on our [177Lu]Lu-DOTATATE patient dataset, using three 3D U-Nets. The skipped 3/4 projections were predicted based on the 1/4 measured projections. Results are evaluated on 5 test patients, with 4 patients used for training and 2 for validation. SpeRF recon results are repeated for comparison with supervised 3D U-Net method

	SpeRF Recon		Supervised 3D U-Net	
	RCR	RCNR	RCR	RCNR
Lesion	**98.3%**	**87.9%**	80.4%	57.4%
Kidney	**97.7%**	**88.0%**	81.9%	63.5%

The extension from NeRF to SpeRF is natural. NeRF was originally designed to render photorealistic novel views of scenes with complex geometries and appearances by representing a scene as a continuous function that outputs radiance in the coordinate space. To learn this continuous representation, an MLP is trained with scene coordinates as inputs and three-channel RGB colors as the training targets. Similarly, in this work, we employed an MLP to learn a continuous representation, but the training targets were defined as single-channel SPECT projection counts. This coordinate-based learning approach operates directly in the projection domain, making it agnostic to the choice of image reconstruction method. It is compatible with a wide range of reconstruction techniques, including model-based image reconstruction (MBIR) and plug-and-play [32] approaches. MBIR methods typically process a complete set of projection views with fewer counts per view, improving image quality and reducing noise by incorporating regularizers and priors. However, these methods often require careful tuning of regularization parameters, which can be challenging. In contrast, SpeRF is tuning-free, as demonstrated by its robust performance across two distinct therapies with significantly different activity distributions in the body.

While SpeRF recon effectively compensates for image quality degradation in sparse view acquisitions, several limitations remain. At a DF of 4, SpeRF recon achieved RCNRs of ~ 80% or higher for all organs and lesions in patient studies, outperforming other sparse view methods (~ 60–70%, Tables 4 and 5). However, at higher DFs, such as DF = 8, we observed reduced activity recovery (Tables 2 and 3). This reduction likely stems from the neural network’s smoothing tendency in high-noise scenarios, where voxel values are averaged due to noise variances. Additionally, the limited training data at high DFs impacts the MLP’s ability to capture finer textures in measurement projections, further reducing activity recovery for small lesions. A similar reduction in activity/count recovery has also been reported in previous studies [2]. Future research could explore the integration of variational inference or generative models to mitigate this smoothing effect and enhance the fidelity of fine details. Another limitation is the computational efficiency of SpeRF, as it currently takes ~ 40 s to synthesize a single projection image on our machine (RTX 4090 GPU + 24-core Intel i9-13900KF CPU + 64 GB of DDR5 memory), making real-time synthesis challenging. Optimizing the implementations, such as through a customized CUDA kernel, could significantly accelerate processing and address this issue.

Shiri et al. proposed a deep convolutional residual neural network-based approach to reduce SPECT acquisition time by predicting full-time and full-view projection images from half-time and half-view projection images, respectively, to maintain reconstruction quality [16]. Similar to [2], training of [16] was conducted in a supervised manner. However, as shown in Table 6, supervised learning methods are less effective when only limited data is available. We investigated the effects of reducing the acquisition time per projection view by applying retrospective Bernoulli Thinning (BerTin) to full-time projection views. Specifically, let $\:Y\sim\text{P}\text{o}\text{i}\text{s}\text{s}\text{o}\text{n}\left(\mu\:\right)$ and define $\:Z$ such that $\:Z|Y=k\sim\text{B}\text{e}\text{r}\text{n}\text{o}\text{u}\text{l}\text{l}\text{i}(1/\text{D}\text{F},\:k)$. It follows that $\:Z\sim\text{P}\text{o}\text{i}\text{s}\text{s}\text{o}\text{n}(\mu\:/\text{D}\text{F})$. In BerTin, each projection event is independently retained with a probability of 1/DF, resulting in temporally subsampled projection images by a DF [33]. These subsampled projection images are then reconstructed using OS-EM. Table 7 presents metrics, including RA and RCNR for BerTin recons across all [177Lu]Lu-DOTATATE patients. BerTin achieves results comparable to Partial. Both methods maintain high RCR values across all DFs, with BerTin achieving ~ 99% or higher for lesions and kidneys even at DF=8. However, RCNR values for BerTin decline significantly at higher DFs, similar to Partial. For example, at DF=8, BerTin achieves RCNR values of 45.2% for lesions and 49.6% for kidneys, indicating substantial CNR degradation.

**Table 7:** Average RCR and RCNR values for lesions and kidneys for BerTin recons, which reduces acquisition time per projection by retaining projection events with a probability of 1/DF using Bernoulli thinning. Results are based on experiments conducted with our [177Lu]Lu-DOTATATE dataset across various DFs. SpeRF recon results are repeated for comparison with BerTin recon

		DF = 2		DF = 4		DF = 8	
		SpeRF	BerTin	SpeRF	BerTin	SpeRF	BerTin
RCR	Lesion	100.6%	**99.6%**	98.3%	**99.2%**	90.3%	**99.1%**
	Kidney	103.9%	**100.0%**	97.7%	**101.4%**	93.0%	**104.8%**
RCNR	Lesion	**88.6%**	80.1%	**87.9%**	60.2%	**73.5%**	45.2%
	Kidney	**92.6%**	80.5%	**88.0%**	63.2%	**76.5%**	49.6%

Although our research was initially focused on Lu-177 SPECT imaging, we expect that our coordinates learning-based self-supervised method could be adapted for use in other low-count applications, where the imaging acquisition could vary between scans and a single pre-trained model may not generalize well. This includes pure *β*--emitters, like Y-90, characterized by a low yield of bremsstrahlung photons for SPECT imaging [34], and therapies with α-emitters, like Ac-225 that use very low activities [3]. Both present inherent low-count imaging challenges that could potentially benefit from our approach. Furthermore, SpeRF could benefit diagnostic SPECT imaging by enabling administration of lower activities, therefore supporting low-dose SPECT protocols that reduce radiation exposure to patients with minimal compromise to image quality.

## Conclusion

This study addresses the challenge of extended SPECT imaging durations under low-count conditions, as encountered in Lu-177 SPECT imaging, by developing a self-supervised coordinate learning approach, namely SpeRF, that efficiently synthesizes skipped SPECT projection views without separate training data. SpeRF enables a significant reduction in SPECT acquisition time by allowing for skipping projection views and using an MLP to synthesize skipped projections, while preserving image quality, as indicated by improved NRMSD in projections and relative CNR in reconstructions compared with other methods for sparse acquisitions, though minor underestimation of count recovery in patient studies is observed. Unlike supervised deep learning-based approaches, this self-supervised method addresses the challenge of limited training data availability commonly encountered in clinical settings. The feasibility for reduction in acquisition time demonstrated in this work is particularly relevant for imaging under low-count conditions and for protocols that require multiple-bed positions.

## Electronic supplementary material

Below is the link to the electronic supplementary material.

Supplementary Material 1

## Data Availability

The datasets generated during and/or analyzed during the current study are available from the corresponding author on reasonable request. Code for reproducing the results will be available at https://github.com/ZongyuLi-umich/ after the paper is accepted.
