# Supplementary material for: Shorter SPECT scans using self-supervised coordinate learning to synthesize skipped projection views
Source: EJNMMI Phys. 2025 May 20;12:47. doi: 10.1186/s40658-025-00762-3 (PMC12092854; doi:10.1186/s40658-025-00762-3)

**Supplementary Fig. 1**. Individual noise-to-activity-recovery curves for six hot spheres at various down-sampling factors.


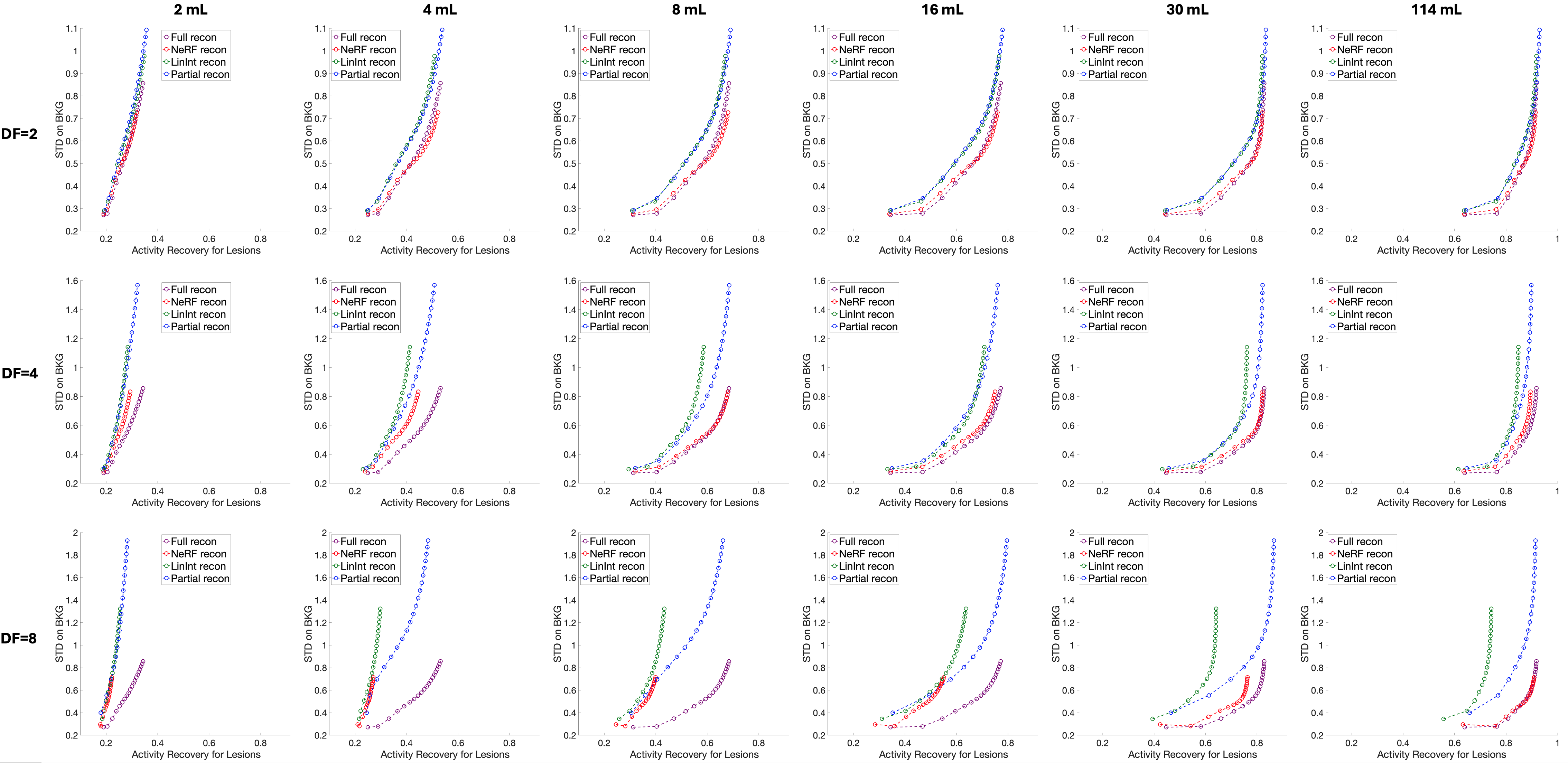

Supplement: Supplementary file 1 — Supplementary Material 1 [file 40658_2025_762_MOESM1_ESM.docx]
